# Artificial Intelligence‐Driven Proteomics Identifies Plasma Protein Signatures for Diagnosis and Stratification of Behçet's Disease

**DOI:** 10.1002/advs.202510061

**Published:** 2025-06-23

**Authors:** Linlin Cheng, Mansheng Li, Zhou Bai, Xiaobo Yu, Wenjie Zheng, Yongzhe Li, Yudong Liu

**Affiliations:** ^1^ Department of Clinical Laboratory State Key Laboratory of Complex Severe and Rare Diseases Peking Union Medical College Hospital Chinese Academy of Medical Science and Peking Union Medical College Beijing 100730 China; ^2^ State Key Laboratory of Medical Proteomics Beijing Proteome Research Center National Center for Protein Science‐Beijing (PHOENIX Center) Beijing Institute of Lifeomics Beijing 102206 China; ^3^ National Center for Clinical Laboratories Institute of Geriatric Medicine Chinese Academy of Medical Sciences Beijing Hospital/ National Center of Gerontology Beijing 100730 P. R. China; ^4^ Department of Rheumatology and Clinical Immunology Peking Union Medical College Hospital Chinese Academy of Medical Sciences & Peking Union Medical College National Clinical Research Center for Dermatologic and Immunologic Diseases (NCRC‐DID) Ministry of Science & Technology State Key Laboratory of Complex Severe and Rare Diseases, and Key Laboratory of Rheumatology and Clinical Immunology Ministry of Education Beijing 100730 China

**Keywords:** Behçet's disease, biomarkers, disease stratification, machine learning, proteomics

## Abstract

The diagnosis of Behçet’s disease (BD) predominantly relies on clinical symptoms, indicating an urgent for identifying potential biomarkers for early diagnosis and disease stratification. We employed an in‐depth proteomics platform based on data‐independent acquisition mass spectrometry (DIA‐MS) and customizable antibody microarray technology, combined with machine learning methods. By analyzing the proteomic data in the training cohort, we trained an XGBoost machine learning model, and validated the model in an independent cohort. The model displayed a favorable performance in BD diagnosis and stratification. In the training set, the area under the curve (AUC) of the diagnostic model was 0.984 with an accuracy of 0.935. In the validation set, the AUC was 0.967 with an accuracy of 0.871. The AUCs for differentiating different severity BD groups ranged from 0.897 to 0.986 in the training set, and from 0.718 to 0.960 in the validation set. Functional analysis indicated that processes such as defense response, protein activation cascade, and complement activation were related to disease severity. Complement C4B was crucial in the protein‐protein interaction network. This study is the first to construct an artificial intelligence‐based BD diagnosis and stratification model, providing potential biomarkers and new strategies for precise diagnosis and treatment of BD. The diagnosis of BD predominantly relies on clinical symptoms, indicating an urgent for identifying potential biomarkers for early diagnosis and disease stratification. We employed an in‐depth proteomics platform based on data‐independent acquisition mass spectrometry (DIA‐MS) and customizable antibody microarray technology, combined with machine learning methods. By analyzing the proteomic data in the training cohort, we trained an XGBoost machine learning model, and validated the model in an independent cohort. The model displayed a favorable performance in BD diagnosis and stratification. In the training set, the area under the curve (AUC) of the diagnostic model was 0.984 with an accuracy of 0.935. In the validation set, the AUC was 0.967 with an accuracy of 0.871. The AUCs for differentiating different severity BD groups ranged from 0.897 to 0.986 in the training set, and from 0.718 to 0.960 in the validation set. Functional analysis indicated that processes such as defense response, protein activation cascade, and complement activation were related to disease severity. Complement C4B was crucial in the protein‐protein interaction network. This study is the first to construct an artificial intelligence‐based BD diagnosis and stratification model, providing potential biomarkers and new strategies for precise diagnosis and treatment of BD.

## Abstract 2

Molecular and functional landscape of BD progression and severity based on in‐depth proteomics. Tree boosting machine learning models with favorable performance in BD diagnosis and stratification. Efficient biomarker panels for BD diagnosis and stratification.

## 1 Introduction

Behçet's disease (BD) is a chronic multisystem inflammatory disorder characterized by a heterogeneous spectrum of clinical manifestations, including mucocutaneous, articular, ocular, vascular, neurological, and gastrointestinal involvement.^[^ ^1^ ^]^ BD predominantly affects populations along the historically known “Silk Route.” While sharing some clinical and pathophysiological features with autoimmune and autoinflammatory diseases, BD exhibits distinct characteristics, such as male predominance, a unique epidemiological distribution, and poor response to anti‐interleukin 1 (anti‐IL‐1) therapies.^[^ ^2^ ^]^ Despite significant research efforts, the pathogenic mechanisms of BD remain largely undefined, involving complex interactions among infectious, genetic, epigenetic, and immunological factors.^[^ ^1^ ^]^

Currently, the diagnosis of BD primarily relies on clinical symptoms. However, nonspecific clinical manifestations and the absence of laboratory biomarkers have significantly hampered its early and accurate diagnosis. In contrast to other autoimmune and autoinflammatory disorders, in which laboratory parameters exhibit critical diagnostic and prognostic value, no laboratory tests, biomarkers are available as diagnostic tools for BD. Thus, there is an urgent need to identify potential biomarkers that can distinguish BD from other autoimmune and autoinflammatory conditions at an early stage, accurately assess disease activity, and monitor or predict disease progression.

Proteomic profiling has emerged as a powerful tool for systematically characterizing dysregulated proteins and activated signaling pathways, which is critical for discovering novel biomarkers. Leveraging an integrated approach combining data‐independent acquisition mass spectrometry (DIA‐MS) with customizable antibody microarray, our previous work identified biomarkers capable of distinguishing BD patients with vascular involvement.^[^ ^3^ ^]^ In this study, we developed a machine learning model using proteomic data from a training cohort to identify biomarkers with both diagnostic and prognostic utility. Model validation in an independent cohort demonstrated comparable performance in diagnosing BD and stratifying patients by disease severity.

## 2 Result

### 2.1 In‐Depth Proteomics Characteristics of BD Patients and HCs

**Figure 1:**
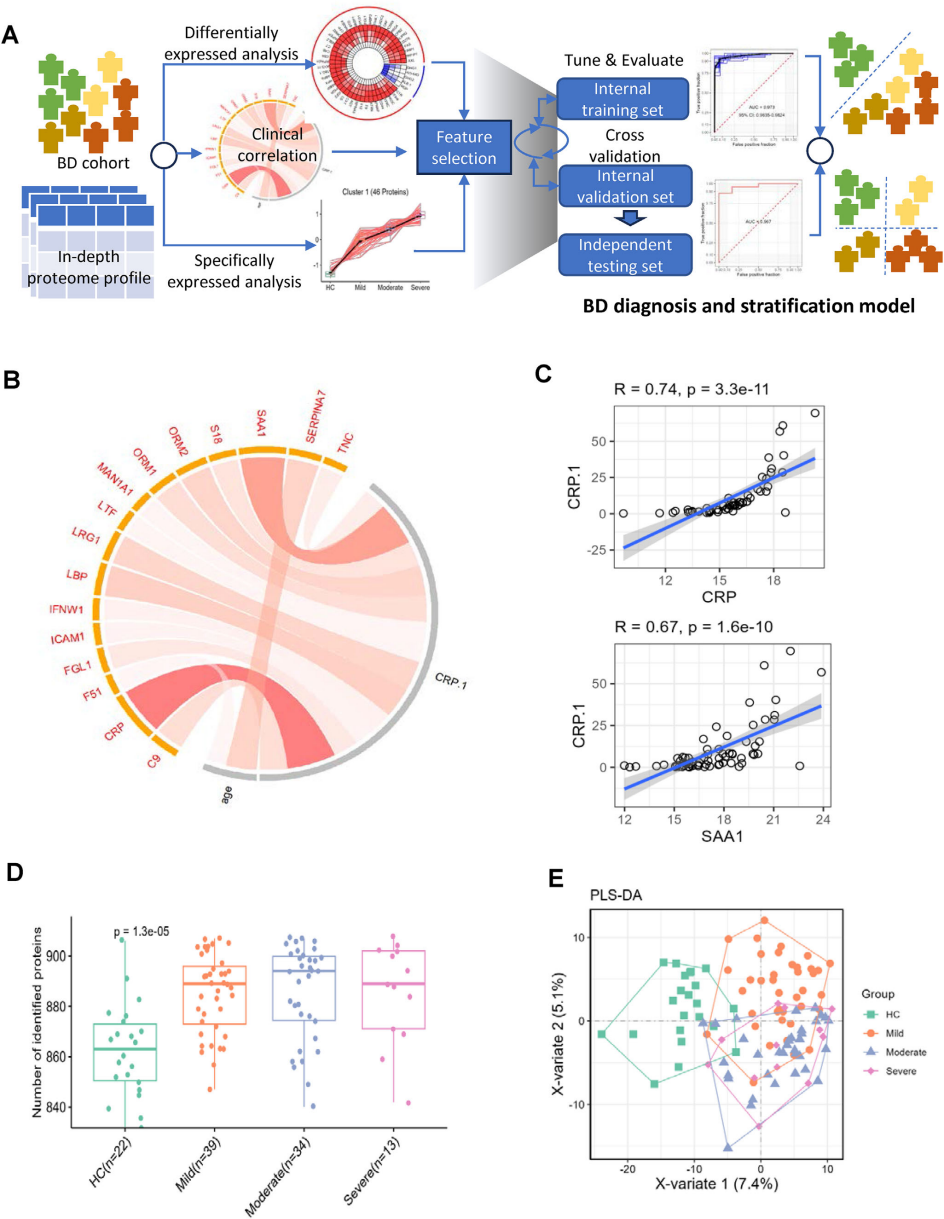
Proteomic profiling of plasma samples from healthy controls and BD patients. A) Study workflow. B) Boxplot displaying the number of proteins identified in plasma samples from healthy controls and BD patients across three severity stages (mild, moderate, and severe). C) Partial least squares‐discriminant analysis (PLS‐DA) of healthy controls and BD patients at three severity stages based on proteomic profiles. D) Correlation network between plasma proteome and clinical data of BD patients. Red and blue circular plots indicate positive and negative correlations, respectively. E) Representative examples of positive and negative correlations between proteins and clinical indices.

The work‐flow of this study is shown in **Figure** **1**A. We first correlated several acute‐phase reactants, including C‐reactive protein (CRP), serum amyloid A (SAA), lipopolysaccharide‐binding protein (LBP) determined by the in‐depth proteomics platform, with CRP (CRP.1) determined by Atellica (Siemens Healthineers) in routine clinical practice. A significant positive correlation between these markers with CRP.1 was identified, confirming the quality and the reliability of the in‐depth proteomics platform (Figure 1B,C).

Overall, a total of 862, 888, 893 and 888 proteins were identified among the HC group and the mild, moderate and severe BD groups (Figure 1D). The Partial Least Squares Discriminant Analysis (PLS‐DA) analysis showed that the HC group (Green color) can be separated from BD patients with different disease severity (orange yellow, blue, pink). In addition, the majority of mild patients was separated from moderate and severe patients. Unfortunately, proteomics analysis failed to distinguish moderate BD patients from severe BD patients (Figure 1E).

### 2.2 Machine Learning‐Driven Diagnostics for Behçet's Disease

**Figure 2:**
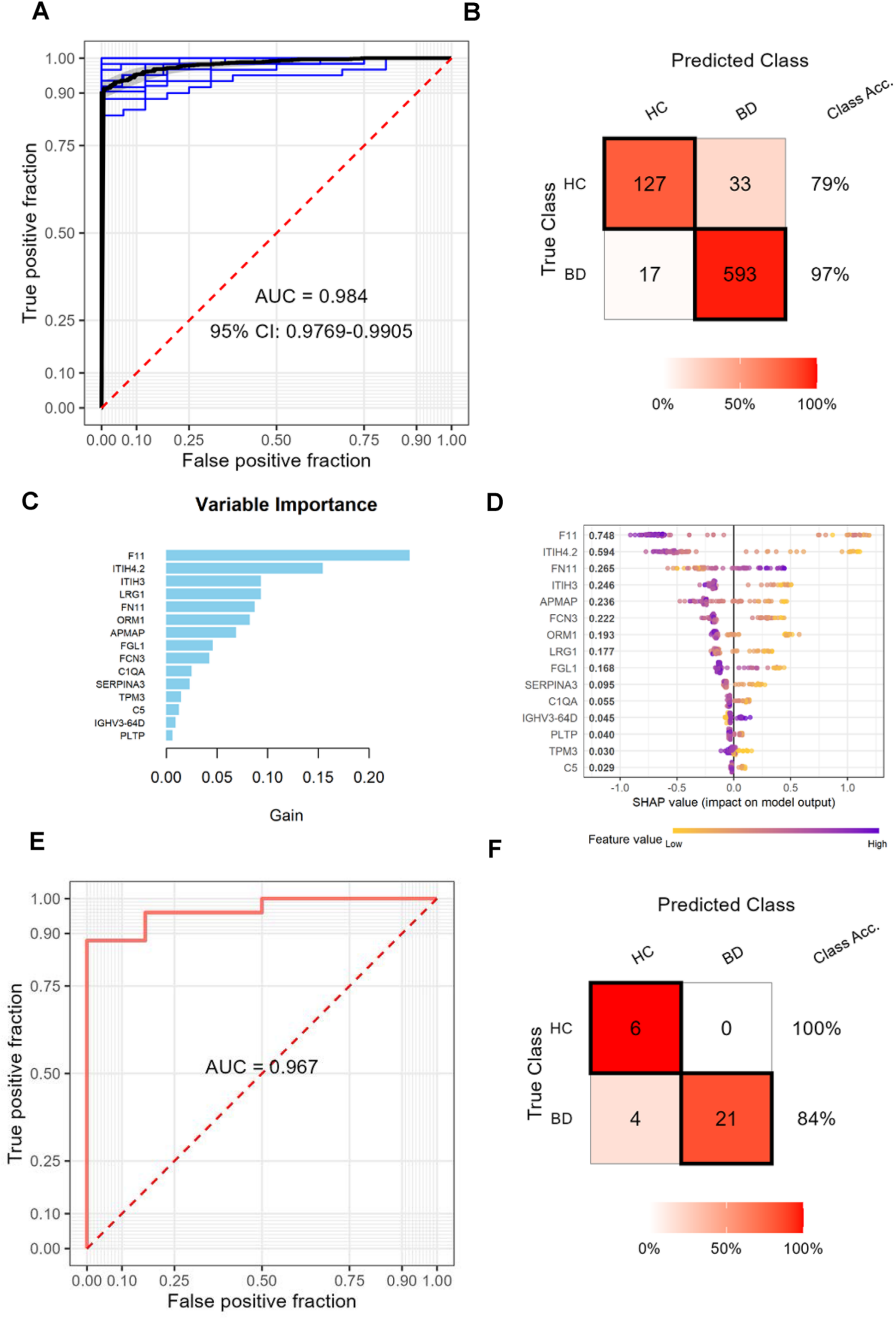
Identification of potential biomarkers for diagnosis of BD patients from healthy controls using machine learning approaches. A) Receiver operating characteristic (ROC) curves for the XGBoost‐based diagnostic model in the training cohort. Blue lines represent cross‐validation results within the training data, with the red dotted diagonal line indicating random prediction performance. B) Confusion matrix depicting model performance for BD diagnosis in the training cohort. Numbers reflect the total counts of correct and incorrect predictions across three repeated 5‐fold cross‐validations with internal train‐test splits. The top 15 feature rankings from the XGBoost classifier, sorted by C) average loss reduction and D) SHAP values, respectively. E) ROC curves for the BD prediction model in the independent testing cohort. F) Confusion matrix illustrating model performance for BD diagnosis in the testing cohort, with counts of correct and incorrect predictions in each category.

To identify proteomic signatures for early BD diagnosis, we applied a machine learning strategy to differentially expressed proteomics data. In the training set, the model achieved an area under the curve (AUC) of 0.984 and an accuracy of 0.935 (**Figure** **2**A,B). Using the XGBoost‐based model, key biomarkers including F11, ITIH4, SERPINA3, APMAP, ORM1, LRG1, ITIH3, FN1, FGL1, FCN3, C1QA, C5, PLTP, TPM3, and IGKV3‐64D were identified as critical variables for BD diagnosis (Figure 2C,D). These findings were externally validated in an independent cohort, yielding an AUC of 0.967 and an accuracy of 0.871 (Figure 2E,F). Collectively, these results highlight the promising potential of AI‐driven proteomics in BD diagnostics.

### 2.3 Functional Analysis of Differentially Expressed Proteins in BD Patients across Different Severity Groups

**Figure 3:**
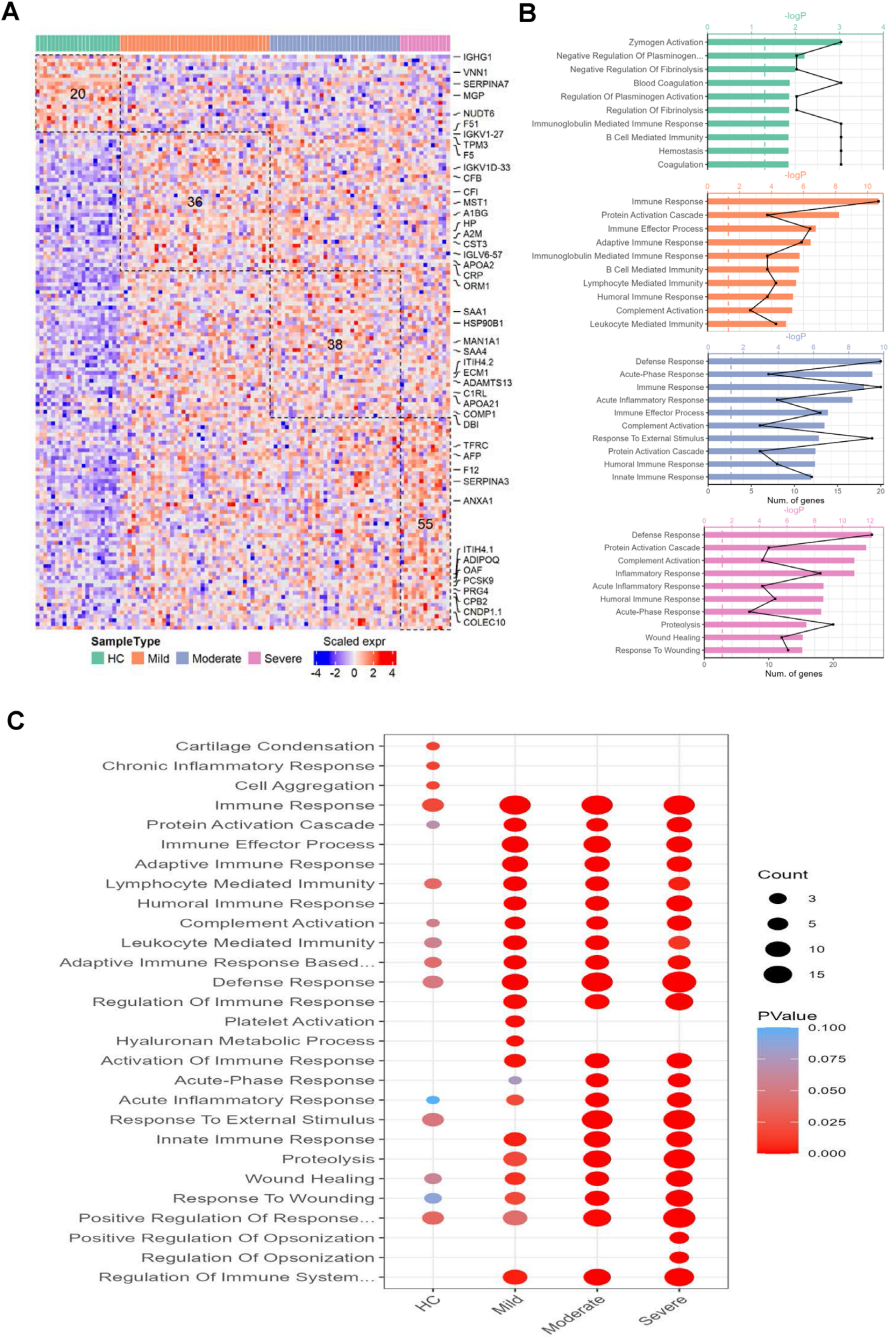
Stage‐specific protein expression analysis in BD. A) Heatmap of proteins identified specifically high‐expressed in plasma samples from healthy controls and BD patients across three severity stages (mild, moderate, and severe), based on log₂‐normalized protein intensities. Red and blue indicate increased and decreased protein abundance, respectively. B) Top 10 enriched GO biological processes for stage‐specific proteins in each BD severity group. C) Cross‐stage comparison of the top 10 enriched GO biological processes in BD stage‐specific proteins. Fisher's exact test was used to determine functional enrichment significance.

Using ANOVA analysis, we identified 20, 36, 38, and 56 proteins significantly overexpressed exclusively in the HC, mild BD, moderate BD, and severe BD groups, respectively (**Figure** **3**A). Functional annotation of biological processes revealed that proteins highly expressed in the mild BD group were enriched in immune response (C1QA, ECM1, IGHV1‐3, TUBB, APOA2, IGHV2‐26, IGKV1D‐33, IGHV1‐18, C3, IGLL5, IGLV1‐51, ADAMTS13, IGKV1‐27, IGLV6‐57, CFHR4, C1RL, LCP1, A2M, PF4, LTF), protein activation cascade (C3, C1QA, CFHR4, FGG, C1RL, FBLN1, A2M), and immune effector response (C3, C1QA, IGLL5, IGHV1‐3, CFHR4, TUBB, C1RL, APOA2, LCP1, IGHV2‐26, A2M, IGHV1‐18). In the moderate BD group, highly expressed proteins were primarily involved in defense response (CRP, ORM1, CFH, C1S, RARRES2, CFI, MST1, KRT1, SAA4, HP, TNC, HPR, IFNW1, KNG1, LOC102723996, C9, SAA1, LBP, APOL1, CFB), acute‐phase response (CRP, ORM1, SAA4, SAA1, HP, HPR, LBP), and immune response (CRP, IGHV3‐43D, CFH, C1S, IGHV3‐11, RARRES2, CFI, MST1, KRT1, IFNW1, ICAM1, VTN, AZGP1, LOC102723996, C9, IGHD, ENPP2, LBP, APOL1, CFB). In the severe BD group, enriched proteins were predominantly associated with defense response (SERPINA3, ITIH4, COLEC10, FCN3, SERPINA1, TFRC, C1R, CFP, C8B, ORM2, CDH5, C5, PRDX5, C7, TIMP1, MBL2, ANXA1, F12, ADIPOQ, SERPINF2, RNASE4, AGT, ATRN, TF, PROC, MASP1) and protein activation cascade with complement activation (FCN3, C5, COLEC10, C7, C1R, F12, MASP1, C8B, CFP, MBL2) (Figure 3B). The contribution of each pathway to different disease severities was further visualized in Figure 3C, which highlights that immune responses, defense response, response to external stimulus, and proteolysis are significantly associated with disease severity. Moreover, these pathways exhibit progressive upregulation with increasing disease severity, and this phenomenon is most pronounced in the severe subgroup when compared to the mild and moderate groups.

### 2.4 Machine Learning‐Driven Stratification of Behçet's Disease Severity

**Figure 4:**
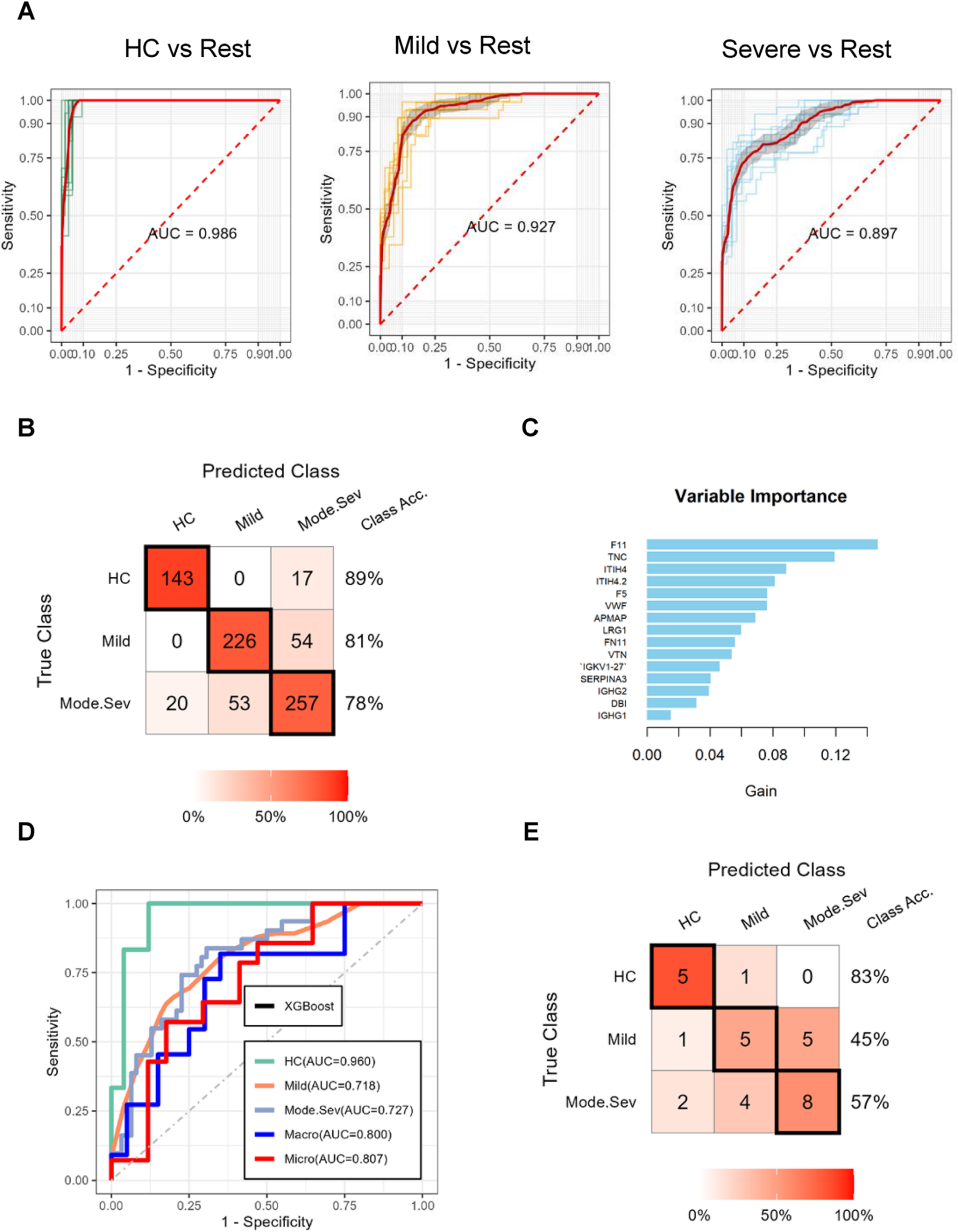
Identification of proteomic signatures associated with BD progression using machine learning approaches. A) Receiver operating characteristic (ROC) curves for the BD progression prediction model in the training cohort. B) Confusion matrix depicting model performance for classifying BD patients across severity stages in the training cohort. Numbers represent counts of correct and incorrect predictions within each category. C) The top 15 feature rankings from the XGBoost classifier for BD stratification, sorted by average loss reduction. D) ROC curves for the BD progression prediction model in the independent testing cohort. E) Confusion matrix illustrating model performance for classifying BD patients across severity stages in the independent testing cohort. Numbers reflect counts of correct and incorrect predictions in each category.

Following differential analysis across disease severity groups, we leveraged AI‐based proteomics to identify circulating protein signatures for BD stratification. The AUCs for discriminating HC from BD, mild BD from other groups (moderate/severe BD and HC), and moderate/severe BD from mild BD and HC were 0.986, 0.927, and 0.897, respectively (**Figure** **4**A,B). Using an XGBoost‐based model, key features including TNC, F11, ITIH4.2, FN11, ITIH4, F5, IGHG2, VWF, VTN, APMAP, IGHG1, LRG1, DBI, IGKV1‐27, and SERPINA3 were identified as critical biomarkers for BD severity stratification (Figure 4C). These results were externally validated in an independent cohort, yielding AUCs of 0.960, 0.718, and 0.727, respectively (Figure 4D,E). Collectively, these findings highlight the potential of AI‐driven proteomics in stratifying BD by disease severity.

### 2.5 Functional Annotation of Deep Plasma Proteomics Underlying Behçet's Disease Stratification

**Figure 5:**
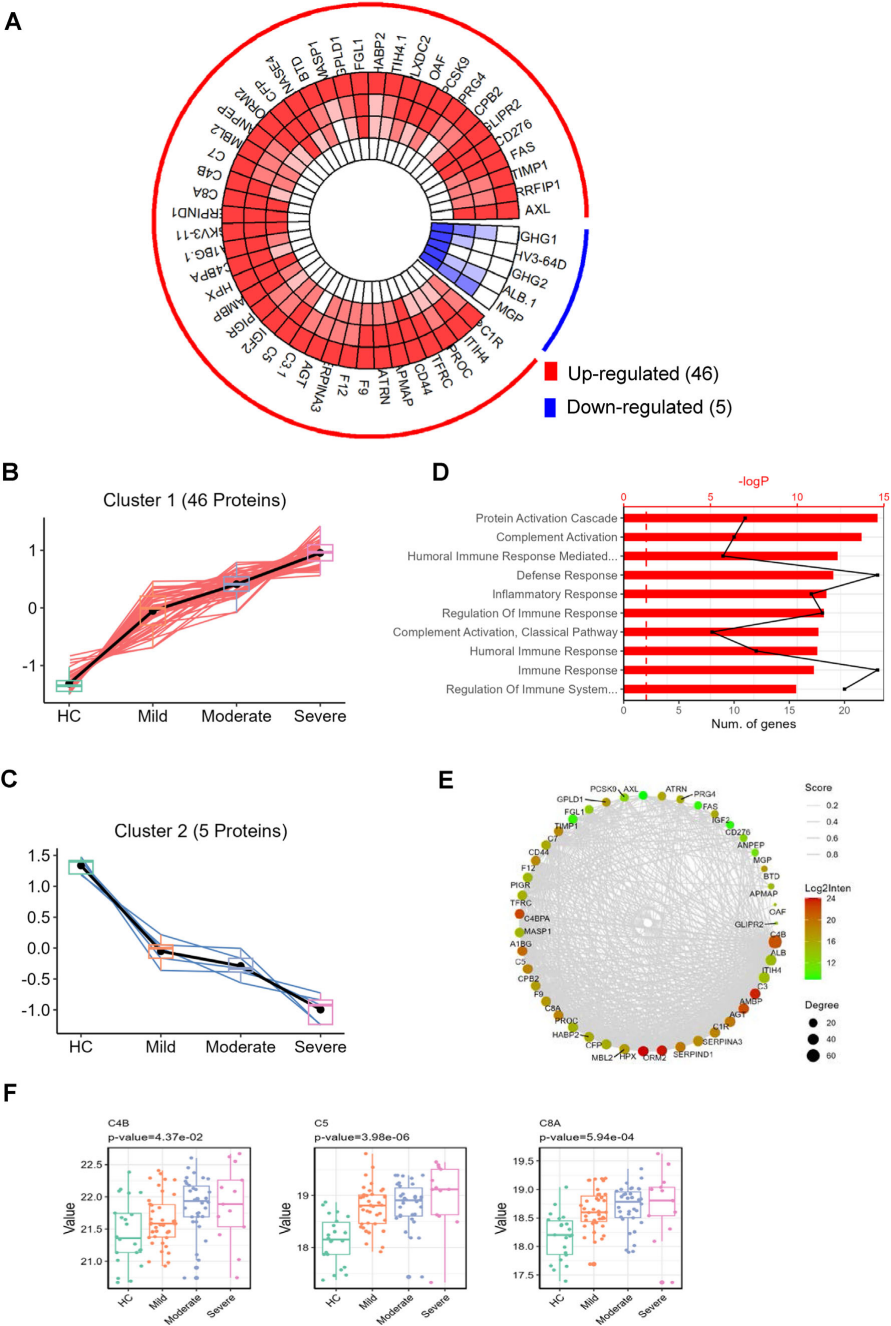
Coexpression patterns of proteins associated with BD progression. A) Circular heatmap (Circos) depicting coexpression networks of proteins correlated with increased BD severity. B,C) Coexpression modules identified by cluster analysis: Cluster 1 (upregulated with increasing BD severity) and Cluster 2 (downregulated with increasing BD severity). D) Top 10 enriched GO biological processes among proteins positively correlated with BD severity. E) Protein–protein interaction (PPI) networks of dysregulated proteins associated with BD progression. F) Boxplots showing expression levels of core complement components (C4B, C5, and C8A) in BD patients, with significant upregulation in severe disease subgroups.

Gene set enrichment analysis (GSEA) was performed to characterize the functional enrichment of proteins implicated in BD pathogenesis. The top three enriched pathways among upregulated proteins in BD versus HCs were *Staphylococcus aureus* infection, platelet activation, and complement and coagulation cascades (Figure S1, Supporting Information). We next investigated pathways associated with disease progression in BD. Single‐sample GSEA (ssGSEA) and expression trend analysis revealed that 55 pathways, including complement and coagulation cascades, antigen processing and presentation, and platelet activation, were correlated with disease progression (Figure S2, Supporting Information). A total of 46 proteins exhibited positive correlations with disease severity, showing progressive upregulation with increasing severity, whereas 5 proteins displayed gradual downregulation patterns with worsening disease (**Figure** **5**A–C). Functional annotation indicated that severity‐associated upregulated proteins were primarily involved in protein activation cascades, complement activation, and humoral immune responses (Figure 5D). Protein–protein interaction (PPI) network analysis identified C4B as the hub protein with the highest degree centrality (Figure 5E). Key complement components, including C4B, C5, and C8A, were significantly associated with higher disease severity in BD (Figure 5F).

## 3 Discussion

BD is a highly heterogeneous disorder in its clinical course, manifestations, and severity.^[^ ^2^ ^]^ Accurate diagnosis remains a significant challenge in clinical practice, particularly for patients presenting with major organ involvement (e.g., vasculitis or neurological symptoms) without oral ulcers or those with isolated vascular thrombosis as the initial manifestation. Moreover, the diagnostic complexities arising from BD's heterogeneity necessitate patient stratification to inform precision medicine strategies. In this proof‐of‐concept study, we highlighted the utility of an AI‐driven approach integrating deep proteomics to identify plasma protein signatures for BD diagnosis and severity stratification, with broader implications for biomarker discovery in other autoimmune and autoinflammatory disorders.

Previous proteomics studies on BD have predominantly focused on the uveitis phenotype, profiling biological samples such as aqueous humor,^[^ ^4^ ^]^ tear,^[^ ^5^ ^]^ plasma,^[^ ^6^ ^]^ urine,^[^ ^7^ ^]^ peripheral blood mononuclear cells (PBMC).^[^ ^8^ ^]^ Two studies investigating the full clinical spectrum of BD identified novel serum proteins and colon mucosa tissue‐expressed proteins that distinguish BD from recurrent aphthous stomatitis (RAS)^[^ ^9^ ^]^ and Crohn's disease,^[^ ^10^ ^]^ respectively, with potential implications for BD diagnosis and therapy. Liu et al. utilized the Olink Immune Response Panel, consisting of 92 immune‐related proteins, to identify BD‐associated proteins and found 43 differentially expressed proteins in BD patients.^[^ ^11^ ^]^ The most significantly downregulated pathways included Toll‐like receptor 9 and NF‐κB signaling, alongside infection‐related pathways such as toxoplasmosis, pathogenic *Escherichia coli* infection, Epstein‐Barr virus infection, HIV‐1 infection, malaria, and tuberculosis, underscoring the critical role of pathogenic infection in BD pathogenesis. Additionally, five machine learning models incorporating immune markers were constructed, with the neural network model (featuring IL10, FCRL3, MASP1, NF2, FAM3B, and MGMT) demonstrating the best performance. However, the analysis was limited to immune‐related proteins, with only a small subset of biomolecules included in the modeling.

In this study, we characterized the full clinical spectrum of BD, including mucocutaneous, gastrointestinal, vascular, neurological, and uveitis phenotypes, and identified 888, 893, and 888 differentially expressed proteins in mild, moderate, and severe BD groups, respectively. Functional enrichment analysis revealed that defense response, protein activation cascades, and complement activation were significantly associated with higher disease severity. Previous studies have highlighted infection—particularly by Herpesviridae family viruses and certain bacteria—as a critical risk factor for BD, alongside genetic susceptibility.^[^ ^12^ ^]^ The complement system is known to play a pivotal role in the immunological control of bacterial infections.^[^ ^13^ ^]^ Through functional annotation and protein‐protein interaction (PPI) network analysis of proteins upregulated in severe BD, we found that complement components dominate the molecular pathways driving BD progression, with C4B emerging as the hub node in the PPI network based on degree centrality.

C4B is a key component of both the classical and lectin complement activation pathways. Synthesized as a single‐chain precursor, it undergoes proteolytic processing to form a trimeric structure (α, β, and γ chains), which provides a molecular interface for mediating interactions between antigen‐antibody complexes and downstream complement effectors. The complement system is essential for eliminating pathogens and infected cells via phagocytosis, cytolysis, and modulation of cellular immune responses.^[^ ^13^ ^]^ For example, C4B covalently binds to pathogens like *Staphylococcus aureus*, enhancing their opsonization and clearance.^[^ ^14^ ^]^ However, excessive complement activation during infection can lead to dysregulated thrombosis, exacerbated inflammation, tissue damage, and irreversible organ dysfunction.^[^ ^13^ ^]^ In BD patients, the significantly elevated C4B levels observed here may therefore contribute to both pathogenic mechanisms and tissue injury. Collectively, these findings suggest that dysregulated C4B‐mediated complement activation could be a key driver of disease severity in BD.

Given the complexity of BD pathogenesis and clinical heterogeneity, a single biomarker is unlikely to capture the full spectrum of the disease. Leveraging proteomic signatures identified in this study, we developed an XGBoost‐based machine learning model for BD diagnosis and severity stratification. XGBoost, an optimized implementation of the Gradient Boosting Machine (GBM), offers high prediction accuracy, efficient parallel processing, robust handling of missing data, and interpretable feature importance metrics.^[^ ^15^ ^]^ These attributes make it a powerful tool for biomedical applications. Our diagnostic model, incorporating 15 plasma proteins, achieved AUCs exceeding 0.95 in both training and validation cohorts. Additionally, we constructed an XGBoost‐based stratification model to discriminate BD severity groups, yielding AUCs >0.8 in the training set and >0.7 in external validation. Consistently, proteins prioritized by both models were significantly enriched in pathways related to infection, inflammation, and coagulation dysregulation.

Notably, APMAP emerged as a key marker in our analysis. This protein facilitates viral entry for multiple pathogens, acting as a critical host factor for polyomavirus (JCPyV) and human cytomegalovirus (HCMV) infections.^[^ ^16^, ^17^ ^]^ Mechanistically, APMAP interacts with HCMV gH/gL glycoprotein complexes under acidic conditions, enabling nuclear translocation of the tegument protein pp65 during early infection.^[^ ^17^ ^]^ Functional studies demonstrated that APMAP knockdown significantly reduced viral infectivity in glial and epithelial/fibroblast cells, with exogenous expression restoring susceptibility.^[^ ^16^, ^17^ ^]^ These findings underscored APMAP's role as a conserved mediator of viral pathogenesis across diverse viral families and cell types.

In our study, FXI emerged as the top‐ranked feature in both diagnostic and stratification models. F11 (coagulation factor XI), a zymogen present in plasma, is a key component of the blood coagulation cascade. Its activated form (FXIa) initiates the intermediate phase of the intrinsic coagulation pathway by activating factor IX. Puy et al. demonstrated that FXIa increases endothelial cell permeability by inducing cleavage of the VE‐cadherin extracellular domain via activation of a disintegrin and metalloproteinase 10 (ADAM10), thus disrupting vascular barrier function.^[^ ^18^ ^]^ Stępień et al. reported associations between higher baseline FXI levels and post‐thrombotic syndrome, venous thromboembolism recurrence, and venous ulcers, which may be attributed to enhanced inflammation and prothrombotic fibrin clot characteristics.^[^ ^19^ ^]^ Notably, no deep‐vein thrombosis cases were observed in an FXI‐deficient cohort, a rate significantly lower than that in population‐based studies.^[^ ^20^ ^]^ These findings suggest a potentially critical role for FXI in BD vasculitis pathogenesis, warranting further functional validation.

The proteins simultaneously identified in both diagnostic and stratification models also include LGR1. A study by Jin's group demonstrated an association between elevated LRG1 levels and increased infarction volume, stroke severity, and poor prognosis in patients with supratentorial cerebral infarction. The investigation further elucidated a potential mechanism by which LRG1 exacerbated cerebral ischemia‐reperfusion injury through upregulation of ALK1 expression, thereby activating the TGFβ‐Smad1/5 signaling pathway to mediate apoptotic cell death and autophagic processes.^[^ ^21^ ^]^ Subsequently, Feng et al. showed that LRG1 promoted apoptosis and autophagy in cardiomyocytes under hypoxic conditions by regulating hypoxia‐inducible factor‐1α (HIF‐1α).^[^ ^22^ ^]^ Additionally, LRG1 serves as an inflammatory biomarker in autoimmune diseases, including pediatric inflammatory bowel disease,^[^ ^23^ ^]^ systemic juvenile idiopathic arthritis^[^ ^24^ ^]^ and rheumatoid arthritis, and can monitor disease activity even in subsets with normal C‐reactive protein (CRP) levels.^[^ ^25^ ^]^

Our study has several limitations that warrant mention. First, while we developed a machine learning‐based diagnostic algorithm, the relatively small sample size may compromise model generalizability. Second, despite the well‐documented clinical heterogeneity of BD, subtype‐specific modeling was hindered by insufficient case numbers across distinct phenotypic categories. Finally, the lack of prospective validation underscores the need for external confirmation in future studies. Given BD's low incidence and indolent clinical course, long‐term longitudinal follow‐up through multicenter collaboration will be critical to validate the biomarker panel's predictive utility.

In summary, we integrated proteomic data from DIA‐MS and antibody microarrays to demonstrate that infection, antigen processing, complement activation, coagulation dysregulation, and immune‐inflammatory responses are central to BD pathogenesis. While microbial infection may serve as an initiating trigger, the crosstalk among subsequent pathophysiological cascades—such as complement‐coagulation interplay—requires systematic investigation. Leveraging these integrated proteomic datasets, we developed two machine learning models for BD diagnosis and severity stratification, both of which demonstrated robust clinical performance in an independent validation cohort. Key biomarkers including FXI, ITIH4, and LRG1 emerged as top‐ranked features in these models. Prospective validation in larger, multicenter cohorts is essential to evaluate the clinical utility of these proteomic models and their potential for guiding precision medicine in BD.

## 4 Experimental Section

### Clinical Cohorts

Demographic features and clinical and laboratory findings of all the individuals in this study were previously described.^[^ ^3^ ^]^ BD was diagnosed based on the 1990 International Study Group criteria^[^ ^26^ ^]^ or the International Criteria for Behçet's Disease criteria,^[^ ^27^ ^]^ as previously described. The severity of BD was assessed by the Krause's score, which grouped as severe (≥7 points), moderate (4–6 points), and mild (<4 points), as previously described.^[^ ^3^ ^]^ The study was approved by the medical ethics committee of Peking Union Medical College Hospital (PUMCH) (JS‐2049).

### Data‐Independent Acquisition Mass Spectrometry (DIA‐MS) Measurements, Antibody Microarray Measurements and Data Processing

DIA‐MS was performed as previously described.^[^ ^3^ ^]^ The raw data files were imported into the Spectronaut pulsar X 12.0 (developed by Biognosys) for analysis using the default settings. The proteins present in plasma were identified utilizing a microarray equipped with antibodies targeting 544 distinct proteins, as previously described.^[^ ^3^ ^]^ The data processing of DIA‐MS measurements and microarray measurements were previously described.^[^ ^3^ ^]^ Briefly, the DIA‐MS data were log‐transformed (base 2) and normalized using the quantile‐based method built in the “limma” R Package across samples. For the antibody array, intensity was determined as the averages of the two technical replicates.

### Enrichment Analysis of Identified Plasma Proteins and BD Stratification Specific Proteins

The gene set enrichment of pathways was performed using R package fgsea (version 1.28.0) based on the KEGG database (https://www.genome.jp/kegg/mapper/color.html). Pathways that were enriched with a *p* value < 0.05 are shown in Figure S1A (Supporting Information). The signaling pathways of all the involved molecules were determined using Pathview (vision 1.42.0). The over representation analysis of biological process were performed with DAVID (https://david.ncifcrf.gov/) and visualized using ggplot2 (version 3.5.0).

### Correlation Analysis between Clinical Index and Differentially Expressed Proteins

The candidate proteins were associated with disease severity if they met either of the following criteria: 1) *p* < 0.05 by the Kruskal–Wallis test with Dunn's multiple comparison post‐hoc tests or one‐way analysis of variance (ANOVA) with Tukey's multiple comparisons test in the comparison of mean protein concentrations among mild, moderate, and severe groups, which shows an upward or downward trend in the HC group and mild, moderate, and severe BD groups; or 2) *p* < 0.05 in the Pearson correlation analysis between protein concentrations and clinic indices (age and CRP).

### XGBoost‐Based Machine Learning Model

Based on the in‐depth plasma proteome from DIA‐MS and antibody microarray experiment, a new XGBoost‐based classifier was developed to diagnose BD cases. The computational pipeline contains the following four steps: i) dataset preprocessing, ii) feature selection, iii) model training, and iv) model evaluation. Particularly, the protein expression data from HC and BD cohort were first log2‐transformed and then quantile normalized. A total of 159 features showing statistically significant between BD and HC were selected for training a XGBoost‐based machine learning model. To train and evaluate the performance of the model, the dataset was randomly divided from the HC and BD cohort into a training set and a testing set. For the training set, cross‐validation was performed by applying a stratified five fold split and repeated three times, resulting in a total 15 internal training/validation splits to ensure robust estimated of model performance. The R package “caret” (v.6.0–94) was used to sample 70% of the HC and BD cohort (HCs, *n* = 16; BD, *n* = 61) as a training set, while the remaining 30% samples as a testing set. The parameters were tuned using a grid search algorithm with 10‐fold cross‐validation implemented in the “caret” package. The XGBoost models were then retrained using the optimal parameters. Finally, the performances of the XGBoost models were further evaluated based on the independent testing sets.

To predict the BD progression, the other XGBoost‐based models were also conducted on BD stage specific proteins with the same strategy. A total of 149 features showing statistically significant and specifically high‐expressed in HC and BD in mild, moderate and severe stage were selected. A training set contained 70% of the HC and BD cohort (HCs: *n* = 16; BD: mild, *n* = 28, and moderate & severity, *n* = 33) and a testing set contained the remaining 30% samples. The corresponding confusion matrices and receiver operating characteristic (ROC) plots were generated to assess performances using the “caret”, “multiROC” (v.1.1.1), “ggplot2” (v.3.5.0) and “plotROC” (v.2.3.0) packages. To enable the clinical performance of the BD diagnosis or progression prediction model, the features were reduced down to ten based on their ranking of importance. Macro‐averaging and micro‐averaging were performed to measure overall model effectiveness across categories.

### Model Performance Evaluation

Three commonly used metrics, accuracy, sensitivity, and specificity, are used to evaluate the performance of BD diagnose and progression prediction model, as presented below by Equations (1), (2), and (3) respectively.

(1) $$\mathrm{Accuracy}=\left(\mathrm{TP}+\mathrm{TN}\right)/\left(\mathrm{TP}+\mathrm{TN}+\mathrm{FP}+\mathrm{FN}\right)$$

(2) $$\mathrm{Sensitivity}=\mathrm{TP}/\left(\mathrm{TP}+\mathrm{FN}\right)$$

(3) $$\mathrm{Specificity}=\mathrm{TN}/\left(\mathrm{TN}+\mathrm{FP}\right)$$

where TP, TN, FP and FN represent true positives, true negatives, false positives and false negatives, respectively. Accuracy, sensitivity and specificity measure the ability to recognize samples, positive samples and negative samples, respectively.

### Statistical Analysis

Statistical analysis was performed using R version 4.3.1 and DIA‐MS and antibody microarray data were performed at the gene level. The differentially expressed proteins between BD and HC were identified using the moderated t‐test implemented in limma with a *p*‐value < 0.05. Differentially expressed proteins by DIA‐MS and antibody microarray were first identified separately and then combined for further analysis. The muti‐group comparison was performed using the Kruskal‐Wallis tests or one‐way ANOVA. The Pearson correlation coefficient was calculated between the protein levels and clinical characteristics.

## Conflict of Interest

The authors declare no conflict of interest.

## Author Contributions

L.C. and M.L. contributed equally to this work and are co‐first authors. Y.L., Y.L., and W.Z. conceived the study. L.C., M.L., and Z.B. performed the experiments. L.C. and M.L. analyzed the data and drafted the manuscript. Y.L., Y.L., W.Z., and X.Y. revised the manuscript. Y.L., Y.L., W.Z., and X.Y. supervised this study. All authors have read and approved the submitted manuscript.

## Supporting information



Supporting Information

## Data Availability

The data that support the findings of this study are available from the corresponding author upon reasonable request.
