# Supplementary material for: Artificial Intelligence‐Driven Proteomics Identifies Plasma Protein Signatures for Diagnosis and Stratification of Behçet's Disease
Source: Adv Sci (Weinh). 2025 Jun 23;12(35):e10061. doi: 10.1002/advs.202510061 (PMC12463117; doi:10.1002/advs.202510061)
Supplement: Supplementary file 1 — Supporting Information [file ADVS-12-e10061-s001.docx]

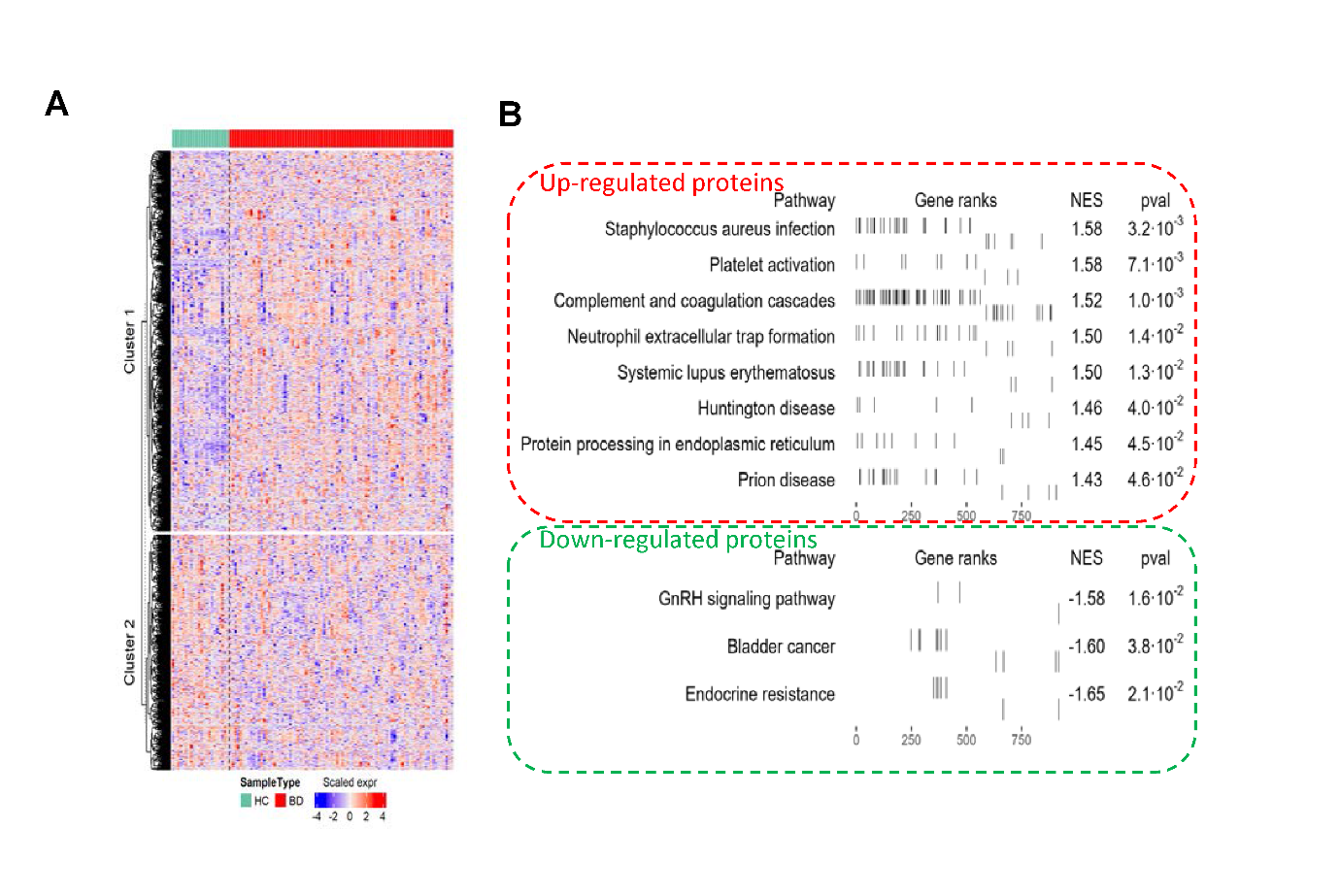


**Figure S1. Gene set enrichment analysis (GSEA) of protein profile of the plasma samples from healthy controls and BS patients.**

**A**. Heatmap show the changes in expression (row z-score normalized) of the whole proteins identified. **B.** GSEA results on the expression of the whole proteins identified providing the signatures evaluated in the context of marker sets representative for hallmarks of BS. The statistical significance (nominal P value) of the enrichment score was calculated using an empirical phenotype-based permutation test procedure.


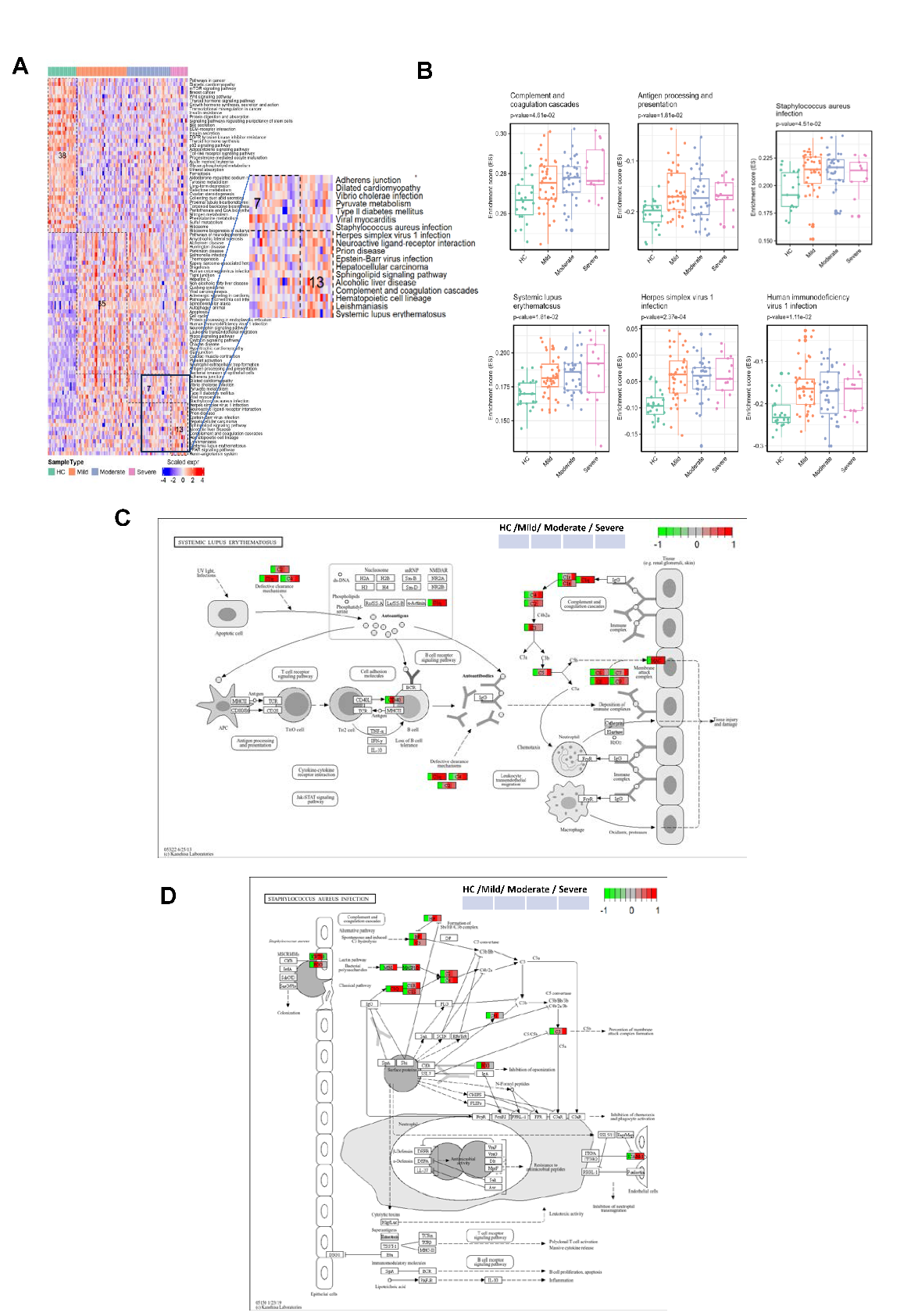


**Figure S2. BS progression-specific activation pathways.**

**A**. Heatmap shows the statistically significant BS progression-specific pathways identified using ssGSEA. **B**. Boxplots show the reprehensive pathways. **C**. BS progression-related proteins in the systemic lupus erythematosus KEGG pathway (hsa05322). **D.** BS progression-related proteins in the staphylococcus aureus infection KEGG pathway (hsa05150).
